# Influence of Forest Disturbance on La Crosse Virus Risk in Southwestern Virginia

**DOI:** 10.3390/insects11010028

**Published:** 2019-12-30

**Authors:** M. Camille Hopkins, Steven D. Zink, Sally L. Paulson, Dana M. Hawley

**Affiliations:** 1 Department of Biological Sciences, Virginia Tech, Blacksburg, VA 24061, USA; mchopkins@usgs.gov; 2 New York State Department of Health, Slingerlands, NY 12159, USA; Steven.Zink@health.ny.gov; 3 Department of Entomology, Virginia Tech, Blacksburg, VA 24061, USA; spaulson@vt.edu

**Keywords:** La Crosse virus, mosquito, chipmunk, invasive species, accessory vectors

## Abstract

Forest disturbance effects on La Crosse virus (LACV) are currently unknown. We determined the abundance of three LACV accessory vectors (*Aedes albopictus*, *Ae. canadensis*, and *Ae. vexans*) and the primary amplifying host (Eastern chipmunk; *Tamias striatus*), and tested for LACV prevalence in both vectors and chipmunks, across a gradient of experimental forest disturbance treatments in southwest Virginia. Forest disturbance significantly affected the abundance of LACV accessory vectors, with a higher abundance on disturbed sites for *Ae. canadensis* and *Ae.* *vexans*. However, there was no significant disturbance effect on chipmunk abundance. Forest disturbance significantly affected LACV prevalence in mosquito vectors, with most (80%) detections on unlogged control sites, which past work showed harbor the highest abundance of the two most common LACV vectors (the primary vector *Aedes triseriatus*, and *Ae. japonicus*). Interestingly, LACV nucleic acid was only detected in *Ae. japonicus* and *Culex pipiens/restuans*, with no detections in the primary vector, *Ae. triseriatus*. In contrast to the vector results, antibodies were only found in chipmunks on logged sites, but this result was not statistically significant. Overall, our results suggest that human LACV risk should generally decline with logging, and reveal the potential importance of accessory vectors in LACV maintenance in Appalachian forests.

## 1. Introduction

Environmental landscape changes can impact vector-borne disease dynamics by affecting insect vectors, vertebrate hosts, or their interaction [1]. Forest vector-borne diseases may be impacted by fragmentation, logging, and deforestation [2]. A well-researched example of the influence of forest fragmentation on vector-borne disease risk is the tick-borne Lyme disease. Forest fragments less than two hectares in size have been associated with an increased density of infected nymphs [3] due to a higher relative abundance of competent vertebrate reservoir hosts in small patches [4,5]. Deforestation, which alters microclimatic conditions, has also been shown to impact mosquito-borne human malaria. In heavily logged landscapes, *Anopheles* mosquitoes and the *Plasmodium* protozoan develop rapidly [6]. This shortened mosquito gonotrophic cycle is associated with increased human biting rates and risk of malaria [7,8]. In contrast to work on human malaria, deforestation is generally associated with a decreased prevalence of avian malaria [9,10,11], though vector abundance was not measured in these studies. Overall, research suggests that the influence of forest disturbance on vector-borne diseases is likely complex and may depend on the ecology of the vectors and vertebrate hosts involved. The impact of forest disturbance on La Crosse virus (LACV) is unknown.

Southwestern Virginia is part of an emerging Appalachian focus of LACV [12,13]. While most cases are subclinical, LACV can cause pediatric encephalitis [14]. This zoonotic mosquito-borne virus is maintained in hardwood forests through the primary LACV vector, *Aedes triseriatus* (Say), by transovarial vertical or intergenerational transmission [15,16], as well as a horizontal (i.e., intragenerational) transmission cycle between mosquitoes and sciurid rodents (especially chipmunks) [17,18]. *Aedes triseriatus* can overwinter the virus in tree holes [19]. While the tree-hole mosquito is the primary vector, two invasive mosquitoes are also contributing to the spread of this disease: *Ae. albopictus* (Skuse) [20] and *Ae. japonicus* (Theobald) [21,22]. In fact, the 2009 LACV detection in *Ae. albopictus* in Texas represented a possible expansion of the LACV range by an invasive mosquito species [20]. There is also evidence that other species, such as *Ae. canadensis* (Theobald) [23], *Ae. vexans* (Meigen) [24] and *Culex* mosquitoes [25,26], may play a role in LACV dynamics. Thus, several Culicidae vectors have been implicated in LACV dynamics. We recently showed that mosquito species diversity in the temperate forest mosquito community in southwest Virginia is largely unaffected by logging and associated forest fragmentation [27]. However, we found that there was an effect of logging on overall mosquito abundance and the abundance of the two most common *Aedes* vectors at our sites, *Ae. triseriatus* and *Ae. japonicus* [27], suggesting population-level effects on vector species that may be critical for resulting LACV risk [28,29].

In addition to effects on LACV vector abundance, logging can also impact the abundance of chipmunks, which serve as amplifying hosts of LACV. There has been some prior work on how forest fragmentation affects Eastern chipmunks (*Tamias striatus*; Linnaeus, 1758), with mixed results. In oak forest in Indiana, chipmunks increased in relative abundance on clearcut sites [30]. In contrast, in West Virginia, *T. striatus* declined in response to clearcutting [31]. Finally, studies in New York and Pennsylvania found no differences in chipmunk abundance between clearcut and mature forest stands [32,33]. Because chipmunks can contribute to the dynamics of several vector-borne diseases, including Lyme disease [34], babesiosis [35], anaplasmosis [36], West Nile virus [37], and La Crosse virus [38], it is particularly important to understand how temperate forest logging influences their abundance and exposure to pathogens.

Here, we seek to understand how logging and associated forest disturbance impact the abundance of accessory LACV vectors and chipmunks, the primary vertebrate amplifying host of LACV [38,39], as well as the prevalence of LACV in mosquitoes and antibodies in chipmunks.

## 2. Materials and Methods

### 2.1. Study Site

Our study sites in Jefferson National Forest in southwestern Virginia are part of a long-term investigation of silvicultural oak regeneration methods on biodiversity, the Southern Appalachian Silviculture and Biodiversity Project (SASAB) [40,41,42]. These oak-dominant (*Quercus* spp.) sites had similar overstory composition, age, and topographic position [40]. Two sites used for this study (Blacksburg 1 and 2; BB1 and BB2, respectively) were located in Montgomery County, VA (37°17′35.73″ N, 80°27′24.63″ W (BB1); 37°18′20.35″ N, 80°26′24.95″ W (BB2)), while a third site (Newcastle (NC)) was located in Craig County, VA (37°27′20.78″ N, 80°23′0.37″ W).

### 2.2. Disturbance Treatments

**Figure 1:**
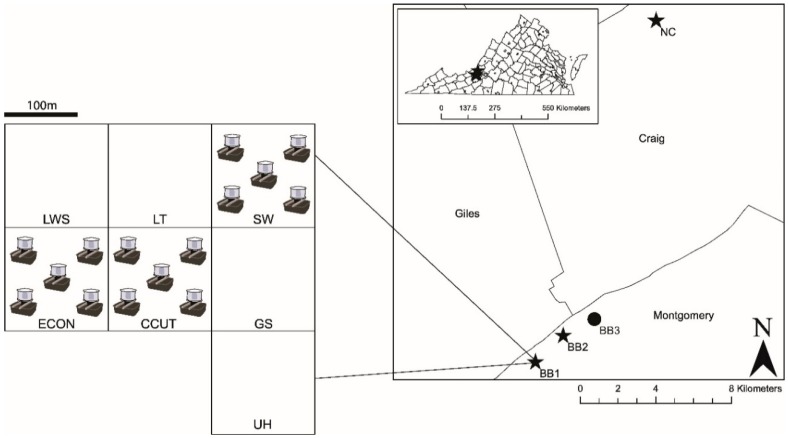
The spatial arrangement of study sites where mosquito abundance and community composition were characterized using gravid traps. The righthand map shows the distribution of the four study sites (BB1 = Blacksburg 1, BB2 = Blacksburg 2, BB3 = Blacksburg 3, and NC = Newcastle) across two counties (Montgomery and Craig), and the inset shows the location of study sites within southwest Virginia. The left-hand figure shows an example layout of the three experimental units sampled at the BB1 study site. Due to experimental randomization, the spatial arrangement of silviculture treatment assignments differed across the three Southern Appalachian Biodiversity Silviculture and Biodiversity (SASAB) study sites (BB1, BB2, and NC; stars). At each SASAB site, three of the seven two-hectare silvicultural treatments (SW = repeat-entry shelterwood, CCUT = clearcut, and ECON = embedded control) were used for this study, but other treatments (LWS = low-leave shelterwood; LT = leave tree cut; GS = group selection; UH = understory herbicide) were shown to illustrate the matrix of habitat within which each experimental unit was embedded. After a minimum 30 m buffer zone was established on each treatment, five infusion-baited gravid traps were placed on each experimental unit, as shown. Gravid traps are not shown to size. Reprinted from [27].

At each of the three SASAB study sites, seven two-hectare experimental units (EUs) were established with no buffer between the units. Silvicultural treatments were randomly assigned to EUs within sites using a fully randomized complete block design (Figure 1). For this study, three two-hectare silvicultural treatments were the focus of mosquito surveillance: repeated-entry high-leave shelterwood (SW) at 0–2 years post-disturbance; single-entry clearcut (CCUT) at 12–14 years post-disturbance; and unlogged control plots embedded in a matrix of surrounding fragmentation created by silviculture treatments, and thus termed “embedded controls” (ECON) at 80–100 years old (Figure 1).

Logging disturbance was examined on a gradient defined by the frequency of harvest and the time elapsed since the last logging event. Embedded control plots had never experienced direct logging but were adjacent to plots that were logged. Clearcut (CCUT) sites were harvested once between 1995 and 1996 [43], and this harvest removed 95% of the basal overstory area. Shelterwood (SW) sites were also logged in 1995–1996, but were only thinned at that time, with 56% of the basal area removed. Just prior to the initiation of this study, SW sites were logged again, with all residual overstory removed [42]. Thus, at the time of this study, SW plots had experienced the most recent disturbance, at 0–2 years post-logging, while CCUT plots were 12–14 years post-disturbance. Thus, we considered our disturbance gradient to be: SW > CCUT > ECON. Accordingly, at the time of the study, the density of overstory trees was highest on ECON sites, intermediate on CCUT sites, and lowest on SW sites [42].

### 2.3. Contiguous Control Sites

Because of the forest fragmentation created by the SASAB experimental design (Figure 1) and associated disturbance to ECON sites (e.g., skid trails, diffuse light from adjacent treatments), an additional non-SASAB study site (BB3) containing two contiguous controls (CCON) equivalent in size to the SASAB EUs was established for the purposes of this study. BB3 (37°18′48.59″ N, 80°2′15.82″ W) was also located in Jefferson National Forest (Montgomery County) for comparison to the embedded control (ECON) at nearby BB1 and BB2. BB3 was approximately 1.8 miles from BB2 (Figure 1). These uncut sites were embedded within large areas of contiguous forest that had not been recently disturbed by harvesting. These stands were dominated by oak (*Quercus alba*, *Q. velutina, Q. prinus*), along with yellow poplar (*Liriodendron tulipifera*). Red maple (*Acer rubrum*) and sourwood (*Oxydendrum arboretum*) were common in the midstory. The ages of the dominant and co-dominant trees in these stands were 100–130 years. Similar to the SASAB sites, the stands were on a south aspect with a moderate slope (J. Overcash, US Forest Service, pers. comm.).

### 2.4. Mosquito Sampling

From late May to September 2008–2010, adult mosquitoes were collected twice a week from infusion-baited gravid traps [44]. Five gravid traps were placed on each EU for mosquito collection (Figure 1). A minimum 30 m buffer zone was applied to each EU to minimize edge effects. After a minimum of 24 h storage in a −80 °C freezer, mosquitoes were identified using morphological keys and pooled into groups with a maximum of 50 females by species, collection site, and date. Male mosquitoes were not counted for this study. Female mosquitoes, which feed on plant sugars and vertebrate blood to obtain nutrients for oviposition, can transmit pathogens to animals and humans [45]. Because important adult taxonomic characters may be damaged or missing after field-collection [46,47], which makes identification difficult, *Cx. restuans* (Theobald) and *Cx. pipiens* (Linnaeus, 1758) mosquitoes were pooled. Such pools will hereafter be referred to as *Cx. pipiens/restuans*. 

### 2.5. Quantitative LACV Real-Time RT-PCR of 2008 Mosquito Pools

Mosquito pools from the 2008 field season were submitted to the Virginia Division of Consolidated Laboratory Services (DCLS) for virus detection. Reverse transcription-PCR was used to determine if this bunyavirus was present on our study sites. One milliliter of bovine albumin diluent (BA-1) [48] was added to each mosquito pool. Mechanical homogenization was performed with a 4.5 mm steel bead; the resultant homogenate was centrifuged for 5 min at 13,500 rpm. Viral RNA was extracted from the supernatant of the homogenized mosquito pools with the QIAamp Viral RNA Mini Kit (Qiagen, Valencia, CA, USA) according to the manufacturer’s instructions. RT-PCR targeting the M segment of LACV was conducted with the QuantiTect probe RT-PCR Kit (Qiagen). We present the threshold cycle (C_T_), defined as the amplification cycle at which the fluorescence increased above the threshold value (i.e., crossing point value).

Samples were tested with the more sensitive primer set (LAC2364, LAC2448; Table 1) for two runs on an ABI PRISM 7000 system (Applied Biosystems, Inc., Foster City, CA, USA). To prevent false positives, samples with a crossing point value were then run on a different machine (the LightCycler 2.0, Roche Diagnostics, Indianapolis, IN, USA), re-extracted twice, and run twice with both LAC2364/2448 and a less sensitive primer set (LAC812, LAC881; Table 1). For each run, 45 amplification cycles were performed. Although no cut-off value was used in 2008, false positives were minimized by running any positive samples for a total of six independent RT-PCRs on two separate machines and using templates from four independent DNA extractions.

Two positive samples from *Cx. pipiens/restuans* (Table 2) collected in 2008 were previously published [26] as part of a study evaluating the vector competence of both *Culex* species for LACV. Similarly, one positive *Ae. japonicus* pool from 2008 (Table 2) and two positive pools from 2009 (see below methods) were previously published as part of a study on emerging field detections of *Ae. japonicus* in the Appalachian region [22]. However, neither study examined these samples in the context of forest disturbance treatment, as we do here. Similarly, those studies did not include the hundreds of negative samples from other vector species, including *Ae. triseratius*, collected as part of this study.

### 2.6. Quantitative LACV Real-Time RT-PCR of 2009–2010 Mosquito Pools

The 2008 results led us to perform viral isolation and quantitative RT-PCR testing of isolates in 2009 and 2010. Mosquito pools were homogenized using previously described methods for LACV isolation [49]. Homogenate supernatant (150 µL) was inoculated onto African green monkey kidney cells (Vero cells, ATCC# CCL-81), incubated at 37 °C and monitored daily for cytopathic effect (CPE). Isolates with marked CPE were harvested and submitted to the Centers for Disease Control and Prevention in Fort Collins, Colorado [20,22,50] or the Wadsworth Arbovirus Laboratories in Albany, New York for molecular testing (see Table 1 and Table 3 for primers). For both laboratories, a sample was considered positive if the C_T_ value was ≤38.

### 2.7. Quantitative LACV Real-Time RT-PCR with Novel Primers

Using the MagMax^TM^ viral RNA isolation kit (Applied Biosystems, Life Technologies, Grand Island, NY, USA) and the Freedom EVO^®^ 150 liquid handling robotic arm (Tecan, Morrisville, NC, USA), RNA was extracted from 100 µL of the submitted cell culture isolate and eluted into 50 µL of elution buffer. The 25 µL reaction mix contained 0.3 µL of 100 µM primer and 0.3 µL of 25 µM probe (Integrated DNA Technologies, Coralville, IA, USA). The thermal cycling consisted of reverse transcription at 50 °C for 2 min, one cycle at 95 °C for 10 min to activate Taq and inactivate the reverse-transcriptase, 45 cycles at 95 °C for 10 s for amplification, and 60 °C for 1 min to read the plate. Amplification and fluorescent detection were performed on the ABI 7500 real-time PCR standard system (Applied Biosystems, Inc., Foster City, CA, USA). For each run, two no template controls were included with the samples. La Crosse virus (LACV/74/NY-M (74-32813)) stock controls were included to control for both the extraction and qRT-PCR. A sample was considered positive if the sample C_T_ value was ≤38, the positive control C_T_ was ≤38, and the negative control C_T_ was >40.

### 2.8. Chipmunk Mark–Recapture Study

In Montgomery County (BB1, BB2, and BB3), a 7 × 7 trapping grid with a 10 m interval was established with 3″ × 3.5″ × 9″ large folding Sherman traps (H.B. Sherman Traps, Tallahassee, FL, USA). Using oats for bait, live trapping was conducted for three consecutive nights at monthly intervals. All captured chipmunks were ear-tagged to allow for mark–recapture estimates of abundance. The following morphometric data were collected from each chipmunk: age class, body and tail length, weight, and reproductive condition. Chipmunks were briefly anesthetized by a licensed veterinarian (M.C.H.) in a small tupperware container using a cotton-ball soaked with isoflurane (Baxter, Deerfield, IL, USA) prior to blood collection. Blood was collected from the orbital sinus or lateral saphenous vein with the collected volume not exceeding 1% of total blood volume [51]. All trapping and handling of small mammals was approved by the Virginia Department of Game and Inland Fisheries (VDGIF # 031626 and 038780) and the Virginia Tech Animal Care and Use Committee (IACUC# 07-083-BIOL and 10-064-BIOL).

### 2.9. Chipmunk Plaque-Reduction Neutralization Test (PRNT) for La Crosse Virus Antibodies

Blood was collected from 52 chipmunk captures. However, four samples were not tested because they were from recaptured chipmunks and nine had inadequate serum volume for PRNT. For recaptured chipmunks, only the last collected serum sample was tested for LACV antibodies. Although traps were placed on the contiguous control sites (BB3), no chipmunks were captured and, therefore, no blood was collected. Serum samples were heat-inactivated at 56 °C for thirty minutes to inactivate viruses and destroy complement. Using BA-1 diluent, sera were initially diluted to 1 in 10 and then titrated by two-fold serial dilutions to 1 in 320 for PRNT assays. LACV-specific neutralizing antibody titers were determined by 90% endpoint PRNT (PRNT_90_). Serum-virus mixture was added to six-well plates with a confluent layer of Vero cells. A 0.5% agarose double-overlay was used and plaques were visualized with neutral red staining in the second overlay, which was applied 48 h after the first overlay [52,53]. Normal guinea pig complement (S1639, Sigma-Aldrich, St. Louis, MO, USA) was added to the serum–virus mixture at an 8% concentration to provide labile serum factor. Each test run was validated with an LACV-specific mouse hyperimmune polyclonal ascitic fluid positive control (World Reference Center for Emerging Viruses and Arboviruses, University of Texas Medical Branch at Galveston, Galveston, TX, USA), normal mouse serum negative control (M5905, Sigma-Aldrich, St. Louis, MO, USA), and an LACV back-titration. Neutralizing antibody titer was expressed as the reciprocal of the endpoint serum dilution that reduced the challenge LACV plaque count by 90% based on the back-titration.

### 2.10. Statistical Analysis

We compared mosquito vector abundance per trap night across forest disturbance treatments using linear mixed-effect models (package nlme). The model with the lowest AIC was selected for this analysis. The model included temporal (year, Julian date) and spatial (study site location: BB1, BB2, NC, or BB3) variables as random effects, with treatment (CCON, ECON, CCUT, or SW) as a fixed effect. This model (AIC: 7081) outperformed models with simpler random effects, which considered location and year (7375), location and Julian date (7109), only location (7391), only year (7402), or only Julian date (7214). We utilized a nested random effects structure with Julian date nested within year and year nested within study site location. The addition of location only slightly improved the model. A likelihood ratio Chi-squared test was used to test for associations between the disturbance treatment of mosquito collection and detection of LACV nucleic acid.

Because of the low density of small mammals, chipmunk abundance was estimated as the minimum number known alive per hectare (MNKA) [54]. MNKA values were calculated based on treatment and year. Therefore, chipmunk abundance (MNKA) across forest disturbance treatments was compared using linear mixed-effect models, with treatment as a fixed effect and year as a random effect. A nonparametric test (Pearson’s Chi-squared test) was used to test for associations between the location of chipmunk capture and presence of LACV antibodies. The prevalence of LACV antibodies across the forest disturbance treatments was compared using the package epiR. The LACV antibody analysis did not include the CCON treatment because no chipmunks were captured on these sites. All analyses were conducted in R version 3.00 (R Development Core Team 2013).

## 3. Results

### 3.1. Mosquito Accessory Vector Abundance

Abundance data for the three most commonly collected vector species at our sites (*Ae. triseriatus*, *Ae. japonicus*, and *Cx. pipiens/restuans*) were previously reported [27], and generally showed significant declines with an increasing degree of forest disturbance (with the exception of *Cx. pipiens/restuans*). Here, we present results for the accessory vectors not examined in prior work.

#### 3.1.1. *Aedes vexans*

**Figure 2:**
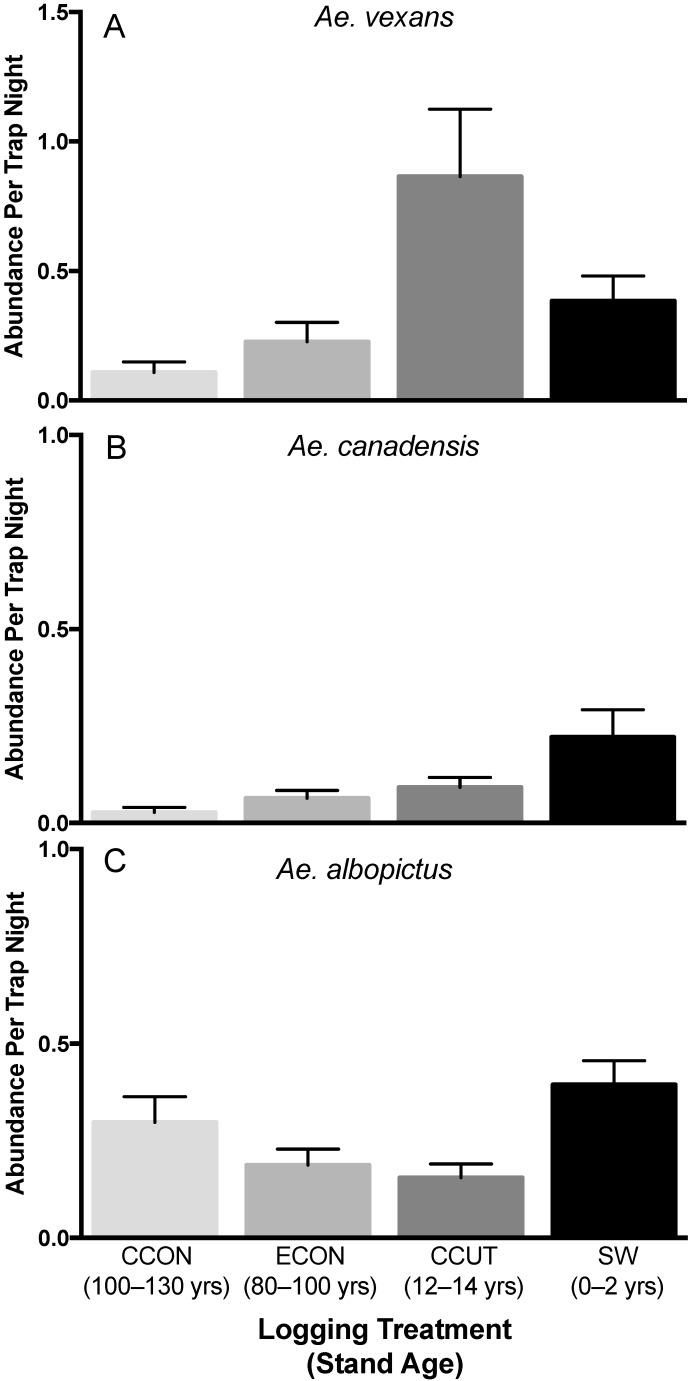
Mean number (± standard error) of female mosquitos from three La Crosse virus accessory vector species (**A**: *Aedes vexans*, **B**: *Aedes canadensis,* and **C**: *Aedes albopictus*) caught per trap-night across forest disturbance treatments, from least (CCON) to most disturbed (SW), as quantified by frequency of harvest and stand age (darkness of shading corresponds to our categorization of the degree of forest disturbance). CCON = contiguous control; ECON = embedded control, CCUT = clearcut, and SW = high-leave shelterwood.

Over three field seasons, 316 *Ae. vexans* adult females were collected. There was a significant disturbance treatment effect on the abundance of this mosquito (F_3,380_ = 4.5, *p* = 0.0042). On average, this vector was most abundant in the clearcut (CCUT) treatment, and was only rarely collected in contiguous control (CCON) sites, which were least disturbed (Figure 2A).

#### 3.1.2. *Aedes canadensis*

Over three field seasons, 79 *Ae. canadensis* adult females were collected. There was a significant disturbance treatment effect on the abundance of this floodwater mosquito (F_3,380_ = 3.5, *p* = 0.016). On average, the shelterwood (SW) treatment (the most recently logged) harbored the highest abundance of this species, and similar to *Ae. vexans*, this species was very rare to absent in contiguous control sites (Figure 2B).

#### 3.1.3. *Aedes albopictus*

Over three field seasons, 191 *Ae. albopictus* adult females were collected. There was a significant disturbance treatment effect on the abundance of this invasive species (F_3,380_ = 6.2, *p* = 0.0004). As with *Ae. canadensis*, the shelterwood (SW) treatment (most recently logged) had the highest abundance of this species, although the next highest abundance occurred on the contiguous control (CCON) treatment, the least disturbed treatment in our study (Figure 2C). Thus, although *Ae. albopictus* was significant impacted by treatment, responses to logging did not appear to be directional in this species.

#### 3.1.4. LACV Mosquito Surveillance

Across all years, LACV nucleic acid was detected in five total pools from two total vector species: *Ae. japonicus* and *Cx. pipiens/restuans* (Table 2). There was a significant effect of treatment on the detection of LACV nucleic acid (χ^2^ = 8.1, d.f. = 3, *p* = 0.044; *n* = 274). The majority of positive samples (4/5, 80%) were in embedded control sites, with the exception of an *Ae. japonicus* pool in the SW. Most positive samples were from sites in Montgomery County, but there was one positive *Ae. japonicus* pool from the Craig County site (NC). 

In 2008, amplification was reproduced in all three positive samples following re-extraction. None of the 2008 positives were successfully confirmed with the LAC812/LAC881 primers. However, these positive samples were all from species (*Ae. japonicus, and Cx. pipiens/restuans*) shown to be competent for La Crosse virus in past work [26,55]. Further, although both positive *Cx. pipiens/restuans* pools had high C_T_ values, several lines of evidence support that those samples are true positives. First, six positive pools of *Cx. pipiens/restuans* were detected in nearby West Virginia using more stringent protocols [26]. Similarly, we previously performed vector competence experiments to demonstrate that both *Culex* species are competent vectors of LACV [26], albeit to a lesser degree than other species. Quantitative RT-PCR revealed no positive pools in 2010, but two positive *Ae. japonicus* pools in 2009 (Table 2).

#### 3.1.5. Chipmunk Mark–Recapture Study

**Figure 3:**
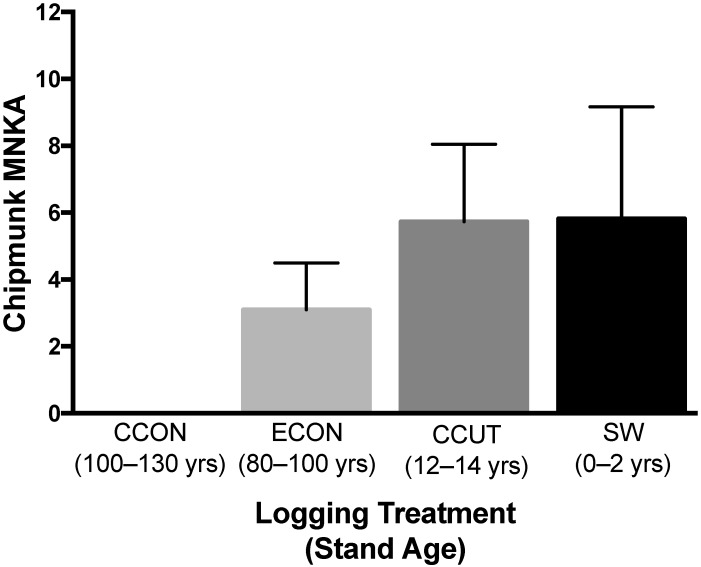
Mean *Tamias striatus* abundance (minimum number known alive per hectare (MNKA) ± SE) across forest disturbance treatments, from least (CCON) to most disturbed (SW) as quantified by frequency of harvest and stand age (darkness of shading corresponds to our categorization of the degree of forest disturbance). CCON = Contiguous control; ECON = Embedded control, CCUT = Clearcut, and SW = High-leave shelterwood.

Forty-eight individual chipmunks were captured over three field seasons. The mean estimate of chipmunk abundance (MNKA) was 3.97 per hectare, with a minimum of zero (CCON) and a maximum of 12 individuals (SW) (Figure 3). There was no significant difference in the MNKA across forest disturbance treatments (F_3,5_ = 3.6, *p* = 0.10).

#### 3.1.6. Chipmunk Plaque-Reduction Neutralization Test (PRNT) for La Crosse Virus Antibodies

Sera from 38 chipmunks collected in Montgomery County (BB1 and BB2) in 2009 and 2010 was tested for LACV antibodies. PRNT (at 90% plaque reduction) confirmed the presence of serum antibodies to LACV in 5 (13%) of 38 chipmunk serum samples. These positive samples were from disturbed sites (SW = 3, CCUT = 2; Table 4). The prevalence of LACV in chipmunks captured on logged sites (pooling SW and CCUT treatments) was 2.75 times (95% CI = 0.17, 44.75) greater than the prevalence in chipmunks captured on the embedded control (ECON) plots. However, there was no significant difference when results were compared across forest disturbance treatments (χ^2^ = 1.376, d.f. = 2, *p* = 0.50). All titers were low (i.e., ≤1 in 20). Most of the positive samples were collected in June, with one of the SW positives collected in late July.

## 4. Discussion

Here, we examined how LACV accessory vector abundance varied with forest disturbance treatment in the southern Appalachians. In contrast to our past work [27] showing that the abundance of the most common LACV vectors on our sites largely declined with forest disturbance, the abundance of LACV accessory vectors largely increased with forest disturbance (Figure 2). LACV nucleic acid detection was greatest on the undisturbed forest sites (Table 2), where the abundance of common LACV vectors was previously documented to be highest [27]. However, seropositive chipmunks were only detected on the two disturbed treatments, although this effect was not statistically significant. Overall, our results suggest that the risk of LACV for humans (as measured by LACV-positive mosquitoes) may be highest in unlogged forest.

Human risk for LACV has been shown to be correlated with the density of *Ae. triseriatus*, the primary LACV vector [56]. This tree-hole mosquito can maintain this bunyavirus in nature through both transovarial and venereal transmission [15,16,57]. Additionally, LACV can overwinter in the diapause eggs of *Ae. triseriatus* [19]. In prior analyses, we found that the abundance of *Ae. triseriatus*, which relies largely on shaded areas and forest trees for oviposition [12,58,59], significantly declined with logging [27]. Here, we showed that La Crosse virus nucleic acid detection in vectors was significantly affected by disturbance treatment, with higher rates of detection on undisturbed sites. In fact, 80% (4/5) of our positive mosquito pools were from embedded control sites (Table 2), while only one positive pool was detected on the shelterwood site. Thus, in agreement with previous work [56], the higher abundance of *Ae. triseriatus* on unlogged forest sites may explain the higher detections of LACV nucleic acid on these sites. However, much to our surprise, none of our detections of LACV were from *Ae. triseriatus*.

Although *Ae. triseriatus* is the primary LACV vector, invasive accessory vectors are increasingly important for LACV risk [20,49]. Two Asian invasive mosquitoes (the Asian rock pool mosquito, *Ae. japonicus,* and the Asian tiger mosquito, *Ae. albopictus*) have become established in the United States [60,61,62,63], including southwest Virginia [64]. These species are known to be competent vectors for LACV [55,65], and can become naturally infected [20,21]. In fact, we detected LACV in three pools of *Ae. japonicus* but not in any pools of the more abundant primary vector, *Ae. triseriatus*, which outnumbered *Ae. japonicus* by 2.6 to 1 at our sites. The Asian tiger mosquito, *Ae. albopictus*, is capable of transovarial LACV transmission [65,66], but the ability of *Ae. japonicus* to vertically transmit this virus is unknown. Our past research together with these results show that invasive vector abundance is differentially affected by logging, which may have important implications for LACV risk. The Asian rock pool mosquito (*Ae. japonicus*) significantly declined with logging [27]. In contrast, here we show that *Ae. albopictus*, known to thrive in sunlit urban areas [10,67], appears to do well on both control treatments and highly disturbed sites, having the highest abundance on the shelterwood (Figure 2). However, we did not detect LACV in any *Ae. albopictus* pools in our sample, and recent modeling work suggests that this species likely does not play an important role in LACV emergence in the Appalachian region [68].

Accessory LACV vectors include well-established invasive and native mosquitoes, in addition to the recent Asian invasive species. Therefore, we also examined the influence of logging on two floodwater mosquitoes, *Ae. canadensis* and *Ae. vexans,* and, in past work, *Culex* species [27]. The La Crosse virus has been isolated from field-collected *Ae. canadensis* [20,23,56,69] and *Ae. vexans* [70]. Although both are considered poor LACV vectors experimentally [71], *Ae. canadensis* has been shown to play a role in LACV dynamics in Ohio and West Virginia [23,56]. We found that the abundance of both *Ae. canadensis* and *vexans* was impacted by logging, with the highest abundance of *Ae. canadensis* found on the shelterwood and the highest abundance of *Ae. vexans* on the clearcut (Figure 2). These patterns are not surprising, because *Ae. canadensis* utilizes a wide range of transient pools, but *Ae. vexans* prefers to lay its eggs in ground depressions in direct sunlight [72]. Although LACV was previously detected in this species in our region [73], LACV was not detected in the *Ae. canadensis* pools on our study sites. However, the low LACV infection rates previously reported [24,56] suggest that it would be difficult to detect this bunyavirus from our small sample size. Finally, our prior analyses examined the effects of logging on *Cx. pipiens/restuans* and found no detectable effects of disturbance on the abundance of this group [27]. Although known to be ornithophilic, there is evidence that *Culex* mosquitoes will feed on mammals [74] and may play a role in LACV dynamics [25,26]. Although both detections of LACV from *Cx. pipiens/restuans* in the current study had relatively high C_T_ values (Table 2), our prior work found that six *Cx. pipiens/restuans* pools collected in West Virginia were positive for LACV, with C_T_ values ranging from 34 to 39, and experimental infections indicated that both species are competent vectors of LACV [26,55]. Further surveillance and research are needed to understand the role of these accessory species in LACV dynamics.

Although vector abundance has been shown to be most important for LACV risk [56], the presence of sciurid rodents is also critical for horizontal transmission [38]. Thus, we examined chipmunk abundance across forest disturbance treatments. While the white-footed mouse (*P. leucopus*), the primary reservoir for Lyme disease, is known to increase in abundance with forest fragmentation [4], the response of *T. striatus* varies [30,31,32,33,75]. Our results indicate that chipmunk abundance (as measured by MNKA) is not significantly affected by forest logging (Figure 3), although it is interesting to note that we did not capture any chipmunks on undisturbed control sites. MNKA is known to underestimate populations [54], so a more extensive mark–recapture study might better elucidate the impact of logging on chipmunks. Future work should include additional trapping methods to assess the abundance of other sciurids that influence LACV dynamics (e.g., squirrels).

In addition to examining chipmunk abundance, we assessed their LACV exposure. Once a chipmunk is infected with LACV, it maintains viremia for an average of 2–3 days [39,76] before developing lifelong immunity evident by antibody levels [17]. Although we found no significant difference in the presence of LACV antibodies across the forest treatments, likely due to small sample sizes, we only detected seropositive chipmunks on disturbed sites (Table 4). These prevalence values were lower than what has been previously reported for chipmunks in endemic areas, where end of season seroprevalence rates ranged from 55% to 100% [18]. The feeding behavior of mosquitoes has to be considered alongside amplifier host antibody prevalence in order to determine resulting risk for LACV horizontal transmission. Generalist vectors such as *Culex* spp. may take more bloodmeals from the abundant rodents on disturbed sites. Alternatively, mammalophilic mosquitoes like *Ae. triseriatus* may dilute LACV by taking a higher proportion of non-amplifier (e.g., deer) bloodmeals on disturbed sites [77,78]. On our sites, there was no detectable difference in deer abundance based on fecal pellet surveys (C.L. Squibb, unpublished data), but the feeding behavior of vectors may be influenced by habitat in addition to simply the abundance of potential bloodmeal hosts.

## 5. Conclusions

Overall, our results together with our past work [27] indicate that the risk of LACV to humans may generally decrease with forest disturbance, although the role of primary versus accessory vectors across gradients of forest disturbance requires further research. The primary LACV vector, *Ae. triseriatus,* and *Ae. japonicus*, the invasive species for which we detected several LACV-positive pools, showed a general decline with logging in our past analyses [27]. Furthermore, the vast majority of our LACV-positive pools were on unlogged, embedded control sites. Thus, together with prior work showing that human risk for LACV is correlated with the density of *Ae. triseriatus* [56], our results suggest that human LACV risk should generally decline with logging. Notably, all of our LACV detections were from species generally considered to be accessory vectors (*Ae. japonicus* and *Cx. pipiens/restuans*). We also examined the abundance of the less common *Aedes* species on our sites and found that the abundance of these vectors generally increased with logging, although LACV was not detected from any of these low-abundance species. While the role of forest disturbance in mediating LACV risk from accessory versus primary vectors remains unclear, our results suggest that, at least in some areas, accessory and invasive vectors may be playing a larger role in the maintenance of La Crosse virus than currently thought [79]. It is critical to continue to fully elucidate the role of vectors other than *Ae. triseriatus*, as well as the role of environmental changes such as logging in the dynamics of LACV transmission in Appalachian regions, which continue to be important emerging foci for this disease.

## Figures and Tables

**Table 1:** Primers used for the amplification of La Crosse virus (LACV).

Year	Laboratory	Primer/Probe Name	LACV M Segment Primer/Probe Sequence (5’→3’)	Source
2008	VA DCLS	LAC836 LP1 LAC812 LF1 LAC881 LR1 LAC2387 LP2 LAC2364 LF2 LAC2448 LR2	CATCCATTCACAGAGTGTGGCACGC TGCAAGCTATGCGGCCTAGT AGCGAGCACCACAGACACAA AATGGGCCAAGTGTGTATAGGAAACCATCA CAATAATTCCGTGTGGTGAACC GACCGATCAGTGCTAGATTGGAA	R. Lanciotti, CDC, pers. comm.
2009	CDC		AGTAGTGTACTACC TTRAARCADGCATGGAA	[50]

**Table 2:** La Crosse virus nucleic acid detection results from mosquitoes collected in southwestern Virginia (2008–2010). Detections were from pools or groups of up to 50 female mosquitoes of the same species caught on the same day and collection site. M = Montgomery; C = Craig; ECON = Embedded control; SW = High-leave shelterwood. n/a = not available. * One *Ae. vexans* pool amplified in one run but because this positive was not reproducible and had a very high C_T_ value (C_T_ = 44), it was assumed to be a false positive and was conservatively excluded from statistical analysis.

Species	Year	LACV + Pools/Total Pools	Mean C_T_ Value	Pool Size	Treatment	County	Month
*Ae. triseriatus*	2008 2009 2010	0/59 0/12 0/11					
*Ae. japonicus*	2008 2009 2010	1/53 2/27 0/16	38 ^a^ 14, 23 ^a^ n/a	22 3, 50 n/a	ECON SW, ECON n/a	M M, C n/a	July July n/a
*Ae. albopictus*	2008 2009	0/10 0/1					
*Cx. pipiens/restuans*	2008 2009 2010	2/64 0/1 0/3	42, 42 ^b^ n/a n/a	3, 7 n/a n/a	ECON n/a n/a	M n/a n/a	July, August n/a n/a
*Ae. vexans*	2008	0/18 *					

^a^ Previously published as part of [22]. ^b^ Previously published as part of [26].

**Table 3:** Primers designed for La Crosse virus M segment amplification.

Primer/Probe	Location in M Segment (GU206142)	LACV M Segment Primer/Probe Sequence (5’→3’)	Primer Size (bp)
F Primer	817	CTATGCGGCCTAGTGTATC	19
R Primer	872	GGAAGTATCATAGCGAGCACC	21
Probe	844	CY5-CACAGAGTGTGGCACGCATTGTGTC-3BHQ_2	25

**Table 4:** La Crosse virus antibody prevalence in chipmunks (*n* = 38) based on plaque-reduction neutralization testing across forest disturbance treatments, from least (CCON) to most disturbed (SW), as quantified by frequency of harvest and stand age. Note that no chipmunks were captured on contiguous control (CCON) sites and thus there were no blood samples to test. A sample was defined as seropositive if there was a 90% reduction in plaques when compared to the LACV back-titration. CCON = Contiguous control; ECON = Embedded control, CCUT = Clearcut, and SW = High-leave shelterwood.

Forest Disturbance Treatment	No. Seropositive/Total Tested	Seroprevalence
Contiguous Control (CCON)	N/A	N/A
Embedded Control (ECON)	0/7	0%
Clearcut (CCUT)	2/14	14.3%
Shelterwood (SW)	3/17	17.6%
Overall Total	5/38	13.2%
